# Acknowledgment to Reviewers of *Brain Sciences* in 2021

**DOI:** 10.3390/brainsci12020171

**Published:** 2022-01-28

**Authors:** Brain Sciences Editorial Office

**Affiliations:** MDPI AG, St. Alban-Anlage 66, 4052 Basel, Switzerland

Rigorous peer-reviews are the basis of high-quality academic publishing. Thanks to the great efforts of our reviewers, *Brain Sciences* was able to maintain its standards for the high quality of its published papers. Thanks to the contribution of our reviewers, in 2021, the median time to first decision was 20 days and the median time to publication was 40 days. The editors would like to extend their gratitude and recognition to the following reviewers for their precious time and dedication, regardless of whether the papers they reviewed were finally published:
Aaron M. JohnsonJody LeonardoAaron MeyerJoel LubarAarti NagayachJoël MacoirAbbas AlZubaidiJoel TalcottAbdellah AdibJohan A. F. KoekkoekAbeer Al-GharaibehJohan KlebergAbhijeet PatraJohan Van Der MeerAbhishek G. SatheJohann SellnerAchilleas AttilakosJohannes KohlAchim BertheleJohannes VorwerkAdam CulbrethJohn A.E. AndersonAdam GeraceJohn ArchibaldAddisson SalazarJohn EllulAdela GoleaJohn GolfinosAdelaide FernandezJohn HeissAdham AtyabiJohn J. HalperinAdina Turcu-StiolicaJohn J. SidtisAdithya SivarajuJohn K. YueAdolfo GarciaJohn KershnerAdoración Carmen Castro GraciaJohn R. ZunigaAdrià ArboixJohn Shelley-TremblayAdrian Elmi-TeranderJohn SteinAfra WohlschlägerJohn-Ross RizzoAfsaneh ShiraniJohn-Stuart BrittainAftab AlamJolande FookenAgata Ando'Jolanta PaukAgata GabryelskaJonas KolbenschlagAgata ZmijewskaJonas WixnerAgnès LacroixJonas-Frederic SauerAgnes Sui-Yin ChanJonatan Ottino GonzálezAgnese CapodieciJonathan D. NeukamAgnese ZazioJonathan P. MillerAgnieszka J. SzczepekJonathan Samuel VogelgsangAgnieszka LandowskaJonathan SchaeferAgnieszka MellerJong Woo LeeAgnieszka NikiforukJoost De VriesAgnieszka WsółJordi Martorell-MarugánAhmad JezziniJordi SorianoAhmed A. HusseinJorge Bosch-BayardAi KoizumiJorge FeitoAine ItoJorge López PugaÁine Ní ChoisdealbhaJorge Matias-GuiuAistė DiržytėJorge OliveiraAistė PranckevičienėJørgen A. RungbyAjay PalJori AkimotoAkhtar AkhtarJos AdamAki HietaharjuJose Antonio Lopez-EscamezAki TakahashiJose BernalAkihiro ShiinaJosé Ignacio Baile AyensaAkiyoshi MatsugiJose L. García-SoidánAkkan AvciJose L. Gómez-UrquizaAkriti SrivastavaJosé Manuel CimadevillaAlan Chuck DorvalJose Pardo VázquezAlan J. NimmoJosé PaviaAlberto BenussiJose Vicente Torres-PerezAlberto DominguezJosee JarryAlberto Javier RamosJosef FinstererAlberto LazarowsykiJoseph K. BelanoffAlberto PisoniJoseph MeyersonAlberto PrioriJoseph R. DuffyAlberto Villarejo-GalendeJoshua C. JuvrudAlberto VogrigJoshua Goh Oon SooAlejandro Galvao-CarmonaJovana BjekićAlejandro MartinJozsef VighAlejandro MartinezJuan Carlos García-MoncóAlejandro Samhan-AriasJuan Carlos López-GarcíaAleksandar VišnjićJuan Lorenzo TerrasaAleksandra SuchaneckaJuan Luis CabreraAleksandra TomićJuan Pedro Martínez RamónAleksei A. StepanenkoJuan Rodríguez MansillaAleksey V. YakovlevJudith BekAleksey ZaitsevJudyta Karolina JuranekAlessandra CostanzaJuhaina Awawdeh ShahbariAlessandra FiorettiJulia A. ScottAlessandra SaporitiJulia CastelloAlessandro AlaimoJulia FöckerAlessandro CastorinaJulia Haba-OscaAlessandro CouyoumdjianJulia M. StephenAlessandro De StefanoJulián J. GonzálezAlessandro RodolicoJulian PackheiserAlessandro SalvalaggioJulian Paul KeenanAlessio FacchinJuliana DushanovaAlessio Paolo BuccinoJuliana YoradnovaÁlex FerrerJulie Boulanger-BertolusAlex KafkasJulie LissAlexander B. KunzJulien ChuquetAlexander G. WeilJulio SecadesAlexander GlushakovJuraj KukoljaAlexander HawlitschkaJürg KesselringAlexander KarabatsiakisJustin W. AndrushkoAlexander MinnaertJwalant MehtaAlexander SavostyanovJyoti MishraAlexander SchützKabirullah LutfyAlexander WeigardKachina AllenAlexandra EconomouKadri KõivAlexandra FietsamKai KammersAlexandra TarabolettiKamil JonakAlexandre Gonzalez-RodriguezKamila RasovaAlexandre KubickiKanna RamaeshAlexandru Florin RogobeteKara JohnsonAlexandru IordanKaren NuytemansAlexey ChubarovKarin DiserensAlexis Estelle WhittonKarin LanderlAlexis R. HarrisKatada SayakoAlfio SpinaKatarina RukavinaAlfonso MastropietroKatarzyna HarezlakAlfredo CaturanoKatarzyna Nowakowska-LipiecAlfredo IacoangeliKatarzyna NowomiejskaAlfredo RaglioKate SimpsonAli RashidiKatharina HüfnerAlice WitneyKatharine C. ReynoldsAlicia Cuesta GómezKatharine HardingAlicja BortkiewiczKatherine CarnelleyAlicja FormaKatherine KoenigAlina Simona RusuKatherine Mercedes HolleranAljoscha ThomschewskiKatherine SherrillAlla BorisyukKathleen A. Welsh-BohmerAlla SalminaKathleen KerrAllan BayatKathrin KollndorferAllan N. SchoreKathrin RothermichAllison GladfelterKathrin WagnerAlthea Z. ValentineKathryn DempsÁlvaro Planchuelo-GómezKathryn DevaneyAlyx TaylorKathryn MuellerAmalia Floriou-ServouKathy DujardinAmanda SeskerKatie HustadAmandine JullienneKatie PetersonAmber GarciaKatlyn BrownAmber JollyKatlyn E. BrownAmbra BisioKatrin T. LübkeAmelia Maria GamanKatrina AlbertAmi CohenKatrina E. FurthAmine GhembazaKatsuhiro MizunoAmira GuirguisKatyayani TatipartiAmit D. GujarKavita SinghAmit PalKayleigh WarringtonAmitai AbramovitchKazuhiro SanoAmjad KhanKazunori HoshinoAmol S. PatwardhanKeisuke SuzukiAna CerveraKeita KamijoAna CicvaricKeith MorrisAna DascaluKeith PecorAna Isabel MolinaKelly HoltAna María González RoldánKelly J. HuffmanAna MuñozKenichi WatanabeAna Ribeiro GonçalvesKenmei MizutaniAna Rodriguez HernandezKenneth HettieAna Sofia CostaKentaro KawabeAna-Maria CazanKevin ChenAnanish ChaudhuriKevin FiteAnastasia StoopsKevin J. BlackAnastasios BezerianosKevin PatersonAnastasiya E. RunnovaKheng Siang NgAnastassia ZabrodskajaKhin ThaAnca Adriana ArbuneKhuen Yen NgAndjela MarkovicKhurshid AhmadAndleeb KhanKi Hwan HwangAndrada IanusKim BulkeleyAndre HussKim L. StansburyAndrea AlexandreKimberly HrehaAndrea BenucciKinga GzieloAndrea BergerKingsley E. AghoAndrea CalvoKiran SandhuAndrea CaraiKiran SapkotaAndrea KwakowskyKiril AlexievAndrea MarcinnòKirrie BallardAndrea MariniKivilcim KilicAndrea MartinuzziKiwamu MatsuokaAndrea MazzatentaKiyotaka NemotoAndrea PaceKlaus JahnAndrea PigoriniKonstantin SlavinAndrea TamásKostromina SvetlanaAndrea VergalloKranti BhavanaAndreanne MichaudKristan LeechAndreas GantenbeinKristina BackerAndreas HøjlundKrupal P. JethavaAndreas IhleKrystyna Bogus-NowakowskaAndreas KyrozisKrzysztof KrystaAndreas SchwarzKwadwo Appiah-KubiAndreas TzschachKyle SueAndree HartantoKyoung-sook JeongAndrei D. SdrullaKyu Young ChoiAndrei DragomirKyuseok KimAndrei SurguchovLahlou GhizlèneAndreia TeixeiraLaia SubiratsAndrej M. SavićLaila CraigheroAndrej PalaLambros MessinisAndrés Da Silva CandalLara A. GreenAndrew E. LittmannLarry RosenAndrew E. PapaleLars KattnerAndrew J. LarnerLasse Dissing-OlesenAndrew K. MartinLasse LindahlAndrew LarnerLaura BaroncelliAndrew Robert BurkeLaura BoschAndrey ChetverikovLaura Del Carmen Sánchez SánchezAndrou AbdalmalakLaura Di GiuntaAndrzej Kazimierz JaworekLaura Fernández-AlacidAndrzej KwolekLaura Fusar-PoliAndrzej PiotrowskiLaura SäisänenAndrzej PolańczykLaura Sánchez-MuñozAndrzej SlominskiLaura SebastianiAndrzej TeisseyreLaura SpinuAndy SiddawayLaura TascónAne Murueta-GoyenaLaura XicotaAneta R. BorkowskaLaurent CarnisÁngel L. GuerreroLayla A. GouldAngel Romero ColladoLazzaro Di BiaseÁngel Romero MartínezLeah FostickÁngel Rosa AlcázarLeanne ElliottAngela Bernabeu SanzLee Byoung-HeeAngela CostabileLeigh Biagio-De JagerAngela DewLeonard AbbedutoAngela GengeLeonardo BrunettiAngela J. FawcettLeonardo Espinosa-LealAngela LukowskiLeonidas StefanisAngela MaffioneLeora YetnikoffAngeliki ZarkaliLeslie CameronAnil AnnamneediLi HuAnil PalaparthiLi SuAnita Cybulska-KlosowiczLiana RomaniukAnke HölligLiangsuo MaAnke SambethLidia GavicAnn HorgasLiisa RaudAnna BelardinelliLijian ZhangAnna BorghiLiliana GarciaAnna Caroline Marques BragaLiliana Ostopovici-HalipAnna CerasoLin ChenAnna ChiariniLina Aj ReissAnna CzlonkowskaLina PalaiodimouAnna FiveashLinda GailīteAnna KlimkiewiczLinda SaraivaAnna Kucharska-NewtonLindsay HalladayAnna LitwiniukLinlin MaAnna ManelisLionel FontanAnna PecchinendaLiron RozenkrantzAnna PetoukhovaLisa A. DaunhauerAnna PittermannLisa ArduinoAnna StarzyńskaLisa GenzelAnna SulekLisa HendersonAnna Ziomkiewicz-WicharyLisa M. GallagherAnnabel D. NijhofLisa Mary SheehyAnna-Leena SirenLisa WagelsAnnalisa LevanteLisa WirzAnna-Lisa SchulerLiudmila LegostaevaAnne Beatty-MartínezLiviu-Adrian CotfasAnne DidierLiye ZouAnnemarie LuikLiz Garcia-PetersonAnnette HandLobato LuisaAnnie VahedipourLogan T. TrujilloAnnika ReinersmannLoredana MarcuAnshul SrivastavaLorena SaelicesAnton FomenkoLorenz S. NeuwirthAntonino NaroLorenzo MagrassiAntonino RaffoneLorenzo PiniAntonino ScibiliaLorenzo RocchiAntonio CataldoLorenzo TonettiAntonio GuaitaLori-Ann R. SacreyAntonio J. Ibanez-MolinaLouis R. NemzerAntonio KokotLuc VallièresAntonio MaffeiLuca BartoliniAntonio NarzisiLuca BruschiniAntonio NicoteraLuca BurroniAntonio Padilha Lanari BoLuca CavaggioniAntonio ParzialeLuca De PalmaAntonio R. Hidalgo-MuñozLuca Di GianfrancescoAntonio RomanoLuca FalciatiAntti KuusinenLuca Giovanni LocatelloAnu PaulLuca Oscar Redaelli De ZinisAnurag ParanjapeLuca RuggieroAraceli Ortiz RubioLucia FuscoArcady MarkelLucilla CardinaliAriel VillagraŁucja BieleninikArmida MucciLucy TroupArnaud GouelleLudise MalkovaArsenio PaezLudmila VīksnaArthur GlenbergLudmiła Zając-LamparskaArtur KrzyżakLuigi Alberto PiniArtur PałaszLuigi SironiArtur Tadeusz KrzyżakLuigi TesioArturo Marti-CarvajalLuigi-alberto PiniArtyom GilLuis E. Bello-EspinosaArvina GrahlLuis Eduardo Coutinho Castelo-BrancoAsad AliLuís Gutiérrez-RojasAshkan EbadiLuis M.T. JesusAshley AchesonLuisa Delgado LosadaAshok PatowaryLuisa Lo IaconoAshu JohriŁukasz SzczukowskiAshwin KumariaLuke J. NeyAstrid D. Adarmes-GómezLu-Ting KuoAstrid ThomasLydia Giménez-LlortAtbin DjamshidianLydia Yahia-CherifAthanasia PatakaLynn WaterhouseAthanasios KoutrasLysianne BeynelAthanasios VourvopoulosMaarten NijkrakeAthina-Maria AloizouMaciej SkotakAtoshi BanerjeeMagda Di RenzoAtsushi NagaiMagdalena Chottova DvorakovaAtul Kumar PandeyMagdalena Jastrzębska-WięsekAudrey ThurmMagdalena KasprowiczAurore PerraultMagdalena LinkeAvi GadothMaggie GuyAxel HuttMagnus LiebherrAxel SteigerMahinda YogarajahAyataka FujimotoMaike DohrnAyumu KonnoMaja Rogić VidakovićBalakrishnan RamasamyMaja ValićBalapal S. BasavarajappaMajed ObaidBanabihari GiriMakoto HosoyaBarbara FivelaMakoto IsozakiBarbara Gili FivelaMałgorzata KujawskaBarbara SpanòMałgorzata LehnerBarbieri ElenaMałgorzata Plechawska-WójcikBaroncelli LauraMamede De CarvalhoBartosz GulaMa'mon M. HatmalBassem HibaManfred BornewasserBeata Labuz-RoszakManish BhomiaBeata Sarecka-HujarManuel FrancoBebiana CondeManuel PereaBegoña EspejoManuel Ramírez SánchezBehnam MolaviManuel SerranoBehnam VafadariManuela PaechterBénédicte M. BatrancourtMaosen WangBeni Gomez-ZunigaMara BreenBenjamin ClarksonMarangelie Criado-MarreroBenjamin CretinMarat MinlebaevBenjamin H. SingerMarawan AbdelBasetBenny BriesemeisterMarc DesforgesBenoit JobinMarc H. E. De LussanetBernardo StutzMarc KerbaBernd FeigeMarc ZanelloBernhard BogertsMarc-André LécuyerBertil RydenhagMarcel BernucciBeth PfeifferMarcel TawkBethany SussmanMarcel Van De WouwBettina M. PauseMarcello GovoniBianca Van KemenadeMarcello MeloneBikash R. SahooMarcello MocciaBilal KhokharMarcin BączykBing-Xing HuoMarco CalabriaBiswajit MaharathiMarco CampenníBjörn MeijersMarco D'AlessandroBłażej RuszczyckiMarco FabbriBoaz BarakMarco FioreBobby StojanoskiMarco GranatoBoel De PaepeMarco IosaBojana DinićMarco R. CelioBonnie FiresteinMarco V. CorniolaBoon-Seng WongMarcos BrioschiBoris De RuyterMarcus D'SouzaBrach PostonMarcus GrueschowBradley EdelmanMarcus KaulBradley R. BuchsbaumMarek CzosnykaBrandon ForemanMarek FraněkBreiffni LeavyMarek KrzystanekBrenton HordacreMargalida Coll-AndreuBrentyn RammMargarida Martins-OliveiraBrian ChicoineMargit PissarekBrian D. AdamsMargot WM De WaalBrian Nils LundstromMaria A. ArvioBrian ParkinsonMaria A. StanleyBrian SkotkoMaria Cristina De ColaBrice T. ClelandMaria Dolores (Lola) De HeviaBriony DowMaria EhnBrittany O’BrienMaria ExindariBrooke-Mai WhelanMaría Felipa SorianoBrunno Rocha LevoneMaria GavriilakiBruno FaconMaria Giovanna BiancoBruno MilletMaria Gómez-CañasBruno SterliniMaria Grazia Razia GrassoByron C. JonesMaria Joana PintoByung-Hoon KimMaria K. JonsdottirCalla M. GoekeMaria LarssonCalvin LuMaria Luisa BringasCameron B. JeterMaría Luisa Delgado-LosadaCameron MangMaria Luisa LorussoCamilla FerrariMaria MilettaCan Ozan TanMaria NiarchouCarine NguemeniMaria Pia BucciCarla LopesMaria Pia GiannoccaroCarlo Alberto ArtusiMaría Pilar Salguero-AlcañizCarlo BuonannoMaria RIta Lo MonacoCarlo Di BonaventuraMaria Rosa Suñer SolerCarlo DoriaMaria RubegaCarlos A. CifuentesMaria SerpenteCarlos J. Hernández-RodríguezMaria SkibińskaCarmen BarbaMaria SofologiCarmen KrewerMaria ValdovinosCarmen López EscribanoMariachiara RicciCarmen López-EscribanoMariacristina MussoCarmen Moret-TatayMarialuisa MartelliCarole SousaMarian SauterCaroline BoxmeyerMarianna SempriniCaroline BrowneMarianna StellaCaroline TiliketeMarianne Skogbrott BirkelandCarolyn LettsMarie Luise SchreiterCaspar StephaniMarie T. KrügerCasper ErkelensMarie-France Champoux-LarssonCatarina GodinhoMarielle BoonenCatarina S. H. JesusMariia MatveevaCaterina LeoneMarilena MarraudinoCaterina Maria GambinoMarina KliuchkoCaterina PiazzaMarina SagudCaterina SquillaciotiMario DalmasoCatherine A. SpannMario MantoCatherine BungenerMario PlaasCatherine PenningtonMario StanzianoCatherine S. HurtMarion TrousselardCeara ByrneMarios AntonakakisCecile Bienboire FrosiniMarisa FilipeCécile DonzéMariusz DruźbickiCecilia BoveMariusz DrużbickiCelia Andreu-SánchezMarjan DrukkerCelia Garcia MaloMark H. MyersCeline PiscicelliMark JonesCeline SaulnierMark Joseph GoldblattCesare ZoiaMark R. DaddsChaklam SilpasuwanchaiMark ReybrouckChaleece W. SandbergMark WagnerChandresh GajeraMark WallaceChang YaramothuMarkus FahlströmChang-Hoon ChoiMarkus Mathaus KoselChanpreet SinghMarkus RiesslandCharalambos K. ThemistocleousMarlise HoferCharles B. DelahuntMarta Aandres-MachCharles CliftonMarta AltieriCharles LeekMarta Anglada-HuguetCharles LenellMarta CampagnoloCharlotte BeaudartMarta HribalChase M. CarverMarta Marszalek-GrabskaChassan ClémenceMarta Morgado CorreiaChenjie ShenMarta TomczykChen-Sen OuyangMarta Vergara-MartínezCheol SongMartin BechChiahao LuMartin DahlChian Ju JongMartin DonnelleyChiara CelataMartina MeszarosChiara FilippiniMary WhitmanChiara MirandolaMaryam ArdalanChiara MoltrasioMarzena MackowiakChiara PagliariMasahiro KawasakiChiara SaraciniMasaki IwasakiChiara SorberaMasanobu IbarakiChiara VillaMasanori YoneyamaChi-Chun HoMasayoshi KuboChiharu TsujiMascha Van’t WoutChiming HuangMassimiliano CastellazziChin-An WangMassimiliano PauChong ChenMassimiliano SommanticoChoon Guan LimMassimo ColettaChrissie ButtsMassimo CossuChrister AhlströmMassimo GrassiChristian BellebaumMassimo MaranoChristian CordanoMassimo PasquiniChristian GoldMassimo TusconiChristian ManciniMathieu PilonChristina Maresch BernardesMathieu SprengersChristina MejersjöMathilde DuchesneChristine BastinMatija MilosevicChristine DuvauchelleMatt J. N. BrownChristof DameMatteo BolognaChristofer LundqvistMatteo CesariChristoph KrausMatteo FabbriChristopher A. WasMatthew E. HirschtrittChristopher StolzMatthew HeathChristopher TenchMatthew J. StainerChristos BakirtzisMatthew McMurrayChristos SidiropoulosMatthias HoenenChun YuanMatthias MulazzaniChun Zhang YangMatthias PriggeCiro ManzoMatthias RiessClaire CuryMatthias SchmitzClaire O'DonovanMatthias WalterClaire PearsonMatti Iso-MustajärviClaire-Marie RangonMaura R. Z. RuzhnikovClark BirdMauricio De Jesus Dias MartinsClaude BesmondMaurizio CasarrubeaClaudia ConsalesMaurizio EliaClaudia Figueroa-RomeroMaurizio MarchesiniClaudia GianelliMauro CostagliClaudia PisanuMauro UrsinoClaudia QuaresmaMax FinkClaudia RicciMax HolzerClaudio TomaMaximiliano A. WilsonClaudio ZuccaM.Carmen Blanco-GandíaClaus Vinter Bødker HviidMd Abdul HakimClayton J. HilmertMd Daud Mohd KhairiClemens BauerMd Jamal UddinClément PimouguetMd Nabiul IslamColin FranzMegan AuldColin HamiltonMegan L. WoodColin J. BarnstableMegan ParkerConcetta De GiambattistaMegan QuimbyCong LiuMeghan MoreanConor J. O'DeaMehmet MahmutConor J. WildMehwish AnwerConstantinos HadjichristidisMei-Chi HsuConxita MestresMei-Hua HallCordula WernerMeira MachadoCorey ClelandMelanie BurkeCorinna BauerMelanie MorrisonCorinna PerchtoldMelinda JacksonCornelia HamannMelissa A. St HilaireCornelia SindermannMelissa MeadeCornelius DonatMelissa RaspaCory MerkelMerce BoadaCosimo IeracitanoMercé FalipCosimo TuenaMercedes SpencerCraig D. WorkmanMerel C. PostemaCraig EvingerMetka NovakCraig H. KennedyMiao YuCraig KelleyMichael C. BurgerCristian DeanaMichael C. KruerCristian E. LeytonMichael Edward SughrueCristian ScheauMichael FreedbergCristina Liébana-PresaMichael MacAskillCristina NunesMichael MacLellanCristina OttavianiMichael OeverhausCristina PolitoMichael PapinuttoCristina Rosell Del ValleMichael SteinbornDagmar Schaffler-SchadenMichael T. KoltzDagmara DimitriouMichael VesiaDaluwathu Mulla Gamage PreethichandraMichael WeichhausDamian VallesMichaela ButrynDamiana ScuteriMichał B. ParadowskiDan RossignolMichal BolaDani TostMichal KlichowskiDaniel BlackburnMichał StrzeleckiDaniel BradfordMichał ZarobkiewiczDaniel BrandeisMichel AudiffrenDaniel C. CastroMichela CandiniDaniel EnglishMichela FerrucciDaniel F. CutlerMichele CiucciDaniel FaboMichele CostanzoDaniel GonzálezMichele FioriniDaniel HolzingerMichele SalemiDaniel J. Van WamelenMichelle BoisvertDaniel MüllensiefenMIchiel ClaessenDaniel SchwarzMickael L. D. DerocheDaniel StjepanovicMIguel Ángel MerchánDaniel VirellaMiguel LázaroDaniela CaldirolaMiguel Reis E. SilvaDaniela De BartoloMihai MusteataDaniela LaricchiutaMihnea-Alexandru GamanDaniela TraficanteMika KallioDaniele Della LattaMike FreedbergDaniele MarzoliMikhail V. VenerDaniele MonzaniMila Dimitrova VulchanovaDanielius SerapinasMilena RaffiDanielle De Souza CostaMilena ŚciskalskaDanielle HitchMilena ŚwitońskaDanielle PhilibertMilind SovaniDarlene A. KertesMilos StanojlovicDarpan ChakrabortyMin Young LeeDavid A. RobbMing YanDavid ArpinMingyu YeDavid Bar-OrMinna TorppaDavid BergeronMinsuk KooDavid D. WardMinyoung KimDavid EilamMira GoralDavid J. AltschulMiranda J. FrancoeurDavid J. HardyMireille BessonDavid M. MeredithMireille VerdierDavid N. RuskinMiren AltunaDavid RyugoMiriam AkkermannDavid S. MooreMiriam BoppDavid T. BundyMiriam BragaDavid T. LiuMiriam HeymanDavid WallonMirjam BrackhanDavide BrottoMiron Bartosz KursaDavide Marco CrociMiroslaw JanowskiDavis FormanMirosław WyczesanyDaya Shankar GuptaMirza PojskicDayna Moya SepulvedaMittal JasoliyaDayu LinMohamed DahmaneDeane AikinsMohamed EmbarkiDebasish RoyMohammad DaneshzandDebo DongMohammad FarhanDeborah Ann HallMohammad Hassan A. NoureldineDeborah FidlerMohammed E. ChoudhuryDebottam SinhaMohammed S. BermoDebra EhrlichMohsen Mosayebi-SamaniDejan BudimirovicMojtaba VaismoradiDejan JakimovskiMolly EricksonDelphine OudietteMomcilo JankovicDeMond M. GrantMonia VagniDenis Delisle RodriguezMonica BucciantiniDenis PeruzzoMónica Muñoz-LópezDenise GobertMonica NizzardoDenise PergolizziMonika Dmitrzak-WęglarzDennis AdjepongMonika LewandowskaDennis Boye LarsenMonika M. Polczynska-BletsosDennis EdlerMonique A. M. SmeetsDerek MitchellMonokesh Kumer SenDezso NamethMontserrat ComesañaDhanesh Sivadasan\t BinduMontserrat ZurrónDhanush HaspulaMonty SilverdaleDhrubajyoti ChowdhuryMootaz SalmanDi ShiMorgan MadecDiana Dow-EdwardsMoritz DannhauerDiana DuroMoses O. SokunbiDiana KimonoMossaed AlyahyaDick TibboelMoussa A. ChalahDiego Fernandez-DuqueMoussa Antoine ChalahDiego ScheggiaMuge KaramanDietmar KrautwurstMuhammad IkramDíez Revuelta ÁlvaroMuhammad Raheel BhuttaDimitrios KatsavelisMukesh DhamalaDimitrios PanagopoulosMuthuraju SanguDimitry VasilyevNabab KhanDina Di GiacomoNachshon KoremDinesh JillellaNadinne RomanDionysios TafiadisNadja FreundDirk K. F. MeijerNagendran MuthusamyDirk SchrieferNagisa SugayaDivya SharmaNaiara DemnitzDmitry O. SinitsynNaiara OzamizDmitry SergeevNamkwon KimDmitry VelmeshevNancy Aaron JonesDominic GaschoNancy CovellDominic StandageNancy FranklinDominic ThyagarajanNaoki YamamotoDominik SaulNaoro TanakaDominique PritchettNaoyuki TakeuchiDona E. LockeNapoleon TorresDonald LobsienNarendra JhaDong Woo KangNatalia Albaladejo-BlázquezDorit MöhrleNatalia Lima MachadoDorota Siwicka-GierobaNatalia MadetkoDorothea FlorisNatalia TrpchevskaDorothee KüemmererNatalie M. ZahrDoug KingNatalie SuffDragan StojanovNataliia KozhemiakoDragica SelakovicNatarajan ManikandanDragos Catalin JianuNatascia De LuciaDragos SerbanNathalia Fernandes FagundesDrozdstoy StoyanovNathalie SchicktanzDulce Maria MinayaNathan M. D’CunhaDuncan StearnsNathan M. PetroDylan S. SpetsNathanael James YatesEberhard UhlNava ZisapelEce BayramNawab DarEdite Teixeira-LemosNazarii KobyliakEdmundo Rosales-MayorNeal RoeseEdoardo MazzucchiNeamtu BogdanEdoardo MonfriniNeepa J. PatelEduardo DovalNeeraj SoniEduardo EuropaNeeraj UpadhyayEdvard EhlerNeil GlossopEdward Baron ShortNeill BroderickEdward GlasscockNell MaltmanEdward MerrillNelson Andrade-GonzálezEdward QuadrosNeven RicijasEdward TronickNicholas B. LarsonEdwin MaasNicholas PintoriEfi KokkotouNichole LighthallEgil NygaardNick DonaldsonEjung MoonNick StrzalkowskiEkaterina DenkovaNicklas Heine StaunstrupElad LaxNicola Di StefanoElena AzziniNicola ModugnoElena D. BazhanovaNicola MontanoElena SalvatoreNicola MontemurroElena SorokinaNicola TambascoElena ZaretskyNicolas GervasiEleni PeristeriNicolas LejeuneEleonora FiorenzatoNicolas PoirelEleonora MascheroniNicole CampeseEleonora OrtuNicole WolffEliana LacerdaNicolo ZarottiEliana RuettiNiels SchillerElicia EstrellaNieves Fátima Oropesa RuizElie MassaadNikita FrolovElisa KallioniemiNikola JajcayElisa LazzariNikolaos GrigoriadisElisa UntiNikolaos SmyrnisElisabet SerratNilo RivaElisabeth Gulowsen CeliusNina HanningElisabeth J. LeehrNina Romanczuk-SeiferthElisabeth Wallhäusser-FrankeNirvana PopescuElisabetta CalettiNitika KumariElisabetta GiorginiNobuhiko HatanakaElisabetta SignorielloNoemi García-RomeroElise BarbeauNoga CohenEliseo Diez-ItzaNorimitsu BanElizabeth A. MartinNorman Scott LitofskyElizabeth CondliffeNounagnon AgbanglaElizabeth De SchauwerNoureddin Nakhostin AnsariElizabeth FoxNozar AghakhaniElizabeth HillNuria Álvarez-SánchezElizabeth M. MartinNuria P. Torres-AguilaElke HattingenNuttida RungratsameetaweemanaEllen Van Der PlasOded KlavirElliot MurphyOksana SintsovaEloy Martinez-HerasOksana SorokinaElse EisingOlaf HaukElvira MaranesiOlaia Lucas-JiménezElżbieta Lorenc-KociOleg SkugarevskyElzbieta Zdankiewicz-ŚcigalaOlga ErmakovaEmanuela CollaOlga IvanovaEmanuela ViggianoOlga Lucia GamboaEmelie LindOlga Mikhailovna BazanovaEmilia I. De La Fuente-SolanaOlga SisoevaEmilia M. GattoOlga V. MartynovaEmilia Zabielska-MendykOlga V. SysoevaEmiliano MerloOliver A. KannapeEmilio PortaccioOlivia AfonsoEmily L. BelleauOlivia RealdonEmmanuel CognatOlivia RemesEmmanuel MulinOlivier BoucherEneko AntónOlivier BraissantEnes AkyuzOlivier PascalisEnkelejda KasneciÓmar Ingi JóhannessonEnrica OlivolaOmid TalakoubEnrico MarcheseOrlando CastellanoEnrico MoroÓscar PozoEnriqueta Canseco-GonzálezOvidiu Popa-VeleaEphrem Takele ZewdieÖzgür OnurEric BravermanPablo José Olivares-OlivaresEric J. HeyerPablo Olivares-OlivaresEric S. DrollettePablo Osvaldo SepúlvedaEric S. PeeplesPablo Sánchez QuinteiroEric W. OttesenPa-Chun WangErica NeriPam HeatonErica SmearmanPanagiotis SimosErich JarvisPankaj GaurErik BehringerPankaj SharmaErika Calvo-OchoaPantelis LioumisErika MolteniPaola Di PietroErin MeierPaola GaldiErmelinda De MeoPaola VenutiErnest TyburskiPaolo BennaErwin MontgomeryPaolo CampioniEslam G. El NebrisiPaolo D'aquilaEstate M. SokhadzePaolo ImmovilliEsteban FridmanPaolo MeneguzzoEsteban Obrero-GaitánPaolo PalmiscianoEsther KuehnPaolo PascoloEstibaliz JarautaParker TichkoEstomih P. MtuiParul VarmaEstrella RausellPascal StammetEttore SilvagniPasquale De FazioEugene AsahchopPasquale ViolaEugene KucharzPatricia J. LardoneEun Sang ChoePatricia Miranda AzpiazuEunyoung HanPatricia SampedroEun-Young ParkPatricia Sampedro PiqueroEva BagyinszkyPatrick AltmannEva DražanováPatrick BrunsEva HavrdovaPatrick CzorlichEva KoetsierPatrick RomaniEva M. Medina-RodríguezPatrick SchussEva SpeckerPatrizia De MarcoEva SzegöPatrizia LoPrestiEvan AntzoulatosPatrycja KleczkowskaEvangelia Maria TsapakisPatty LeijtenEvgeniya V. EfimovaPaul BeachEvgeny BlagovechtchenskiPaul E. EngelhardtEvgeny ErmakovPaul EslingerEwa LitwaPaul FillmoreEwa ZasadzkaPaul HwangEwald NeumannPaul J. ZakEwelina Elert-DobkowskaPaul NyquistFabian HeroldPaula Fernández-PiresFabienne PicardPaula HoffmanFabiola SpolaorPaula RegoFabrice ArcizetPaulo PiresFabrizia FestantePaulo R. B. FernandesFakhri ShafaiPaweł BorkowskiFangyi GuPaweł KrukowFariborz GhaffarpasandPaweł WańkowiczFarzad Vasheghani FarahaniPedro BritesFausta LuiPedro CantistaFayaz Rahman KhanPedro CastroFco J. PeralesPedro Gómez-VildaFederica ScarpinaPedro J. García RuizFederico BilottaPedro PestanaFederico Paolini PaolettiPegah SarkheilFelice GiangasperoPêgo José MiguelFelix ArltPeka ChristovaFelix SiebenhühnerPengmin QinFeng XuePentti HaikonenFengping DongPerrine BrazoFerdinando SartucciPeter A. GoldsteinFergus CraikPeter B. RosenquistFernanda TroiliPeter De LissaFernando CardonaPeter DourisFilipa I. BaptistaPeter E. WaisFilippo CieriPeter HowellFilippo MuratoriPeter KarachunskiFinisguerra AlessandraPeter MayneFiona McNabPeter WitzgallFiras KobeissyPetra AmchováFlaminia FranchiniPetra PohlFlavia CardiniPetrilla JayaprakashFlora GuerraPhilip C. De Witt HamerFlore DepeintPhilip HazellFlorence VorspanPhilip Robert HollandFlorent CarsuzaaPhilipp GuldeFlorian GesslerPhilipp SämannFlorian HeilmannPhilippe TausskyFlorian KattnerPhyllis K. SteinFlorin TibuPier Nicola SergiFlorinda FerreriPierluigi ZoccolottiFrances A. ConnersPierpaolo CroceFrancesca CucinottaPierpaolo IodiceFrancesca Felicia OpertoPierre BessonFrancesca FerroniPierre-Pascal Lenck-SantiniFrancesca ManniPierre-Paul VidalFrancesca StrappiniPietro MuratoriFrancesca TrojsiPietro SpataroFrancesco CacciatorePilar Pérez-RosFrancesco CertoPing-Chieh PaoFrancesco CoreaPiotr AlsterFrancesco CorrivettiPiotr ChmielarzFrancesco GaziaPiotr KrutkiFrancesco GuzziPiotr SobaniecFrancesco MeliPiotr SuffczyńskiFrancesco OrziPiotr WlaźFrancesco Paolo BusardòPiril HepsomaliFrancesco PradaPizza Ka Yee ChowFrancesco RuotoloPratheepa RasiahFrancesco SansonePrisco MarinaFrancesco SignorelliProsper N'GouemoFrancisco Alcantud MarínProspero CivitaFrancisco BarcelóPrzemysław TomasikFrancisco Gonzalez FernandezPuneet BaggaFrancisco Javier BadesaR. James R. BlairFrancisco Luna-PerejónRachel Bar-ShavitFrancisco Murillo-CabezasRachel Rac-LubashevskyFrancisco Nieto EscámezRadek PtakFrancisco Velasco-AlvarezRadosław ZajdelFranck G. SturtzRadu CiorapFranco GemignaniRadwa BadawyFrank BesagRafał Białynicki-BirulaFrank HsuRafał GerymskiFrank KnoefelRafał MorgaFranklin A. MardenRafał PolowyFranz MarxreiterRafeed AlkawadriFranziska KnolleRaffaele MazziottiFranziska LabrenzRaffaele OrnelloFred TravisRaghavan GopalakrishnanFredric SchifferRaha PazokiFrieda MatthysRahul MittalFriedemann MüllerRaili RiikonenFrigerio AlessandraRaimund HelbokFrigyes Samuel RaczRainer Rubira-GarcíaFrouke HermensRajan Deep AdhikariFukuda MitsuhiroRalf BraunGaayathiri JegatheeswaranRalf VeitGabriel Natan PiresRalph D. HectorGabriel RecchiaRama Sashank MadhurapantulaGabriela C. ZaharieRamachandran PrakasamGabriela-Dumitrita StanciuRamesh Kumar MuralimanoharGabriele CiprianiRami DiabGabriele MorucciRamón López-HigesGabriele NibbioRana El RawasGabriella TamburroRandall McKinnonGagan Deep SharmaRandi Dovland AndersenGaku YamanakaRandi Von WredeGale LucasRanran SongGang ChenRao FuGarrett CardonRaphael FargierGarrett M. HesterRaphael HerrGary DonohoeRaphael WurmGary SteinmanRaquel BoiaGaurav GhagRaquel Leirós-RodríguezGaurav KumarRaquel OliveiraGauthaman SukumarRasoul HekmatiGayatri DeviRaul NavarroGema Pérez-RojoRaul ReinaGemma PastorRaúl Soto-CámaraGemma PratRaúl Tárraga MínguezGemma Van RuitenbeekRavi KudesiaGennaro AulettaRaymond Kai Yu TongGennaro RuggieroRebecca BaillieGeorg AdlerRebecca BöhmeGeorge K. E. UmanahRebecca GordonGeorge L. HinesRebecca J. LeppingGeorge M. OpieRebeen Ali HamadGeorge P. ParaskevasReidar P. LystadGeorgios KotzalidisRemington L. NevinGernot HortsmannRenata Del GiudiceGerold BesserRenata Graciele ZanonGerrit KentnerRenata PecoticGert De GraafRenato De-filippisGiacomo BoffaRenske I. WadmanGiacomo InseroRiccardo MancaGiacomo NovembreRichard A. GrünewaldGiada LettieriRichard C. DowellGiampietro AlbertiRichard D. FoustGian Carlo BellenchiRichard FunkGian Daniele ZanninoRigmor Højland JensenGian Marco DumaRima KregždytėGian Marco MarzocchiRishi SharmaGian Maria GaleazziRisto KauppinenGianina Teribele VenturinRitesh RamdhaniGianluca Di FlumeriRob P. W. RouhlGianluca EspositoRobert B. PernaGianluca MalatestaRobert BalshawGianluca SaettaRobert D. HendersonGiannicola IannellaRobert DickinsonGil SuzinRobert Emmett KellyGilliard LachRobert HoerrGinny PalegRobert J. DaweGioele GavazziRobert SpangGiorgia CommitteriRobert SteinbachGiorgia ConaRoberta AgabioGiorgio MaggioniRoberto AltieriGiovanna BorrielloRoberto EspositoGiovanna MontanaRoberto GuidottiGiovanni DelnevoRoberto PalumbiGiovanni Freitas GomesRoberto ZefferinoGiovanni LaviolaRobrecht P. R. D. Van Der WelGiovanni MirabellaRocco HaaseGiovanni PellegrinoRochelle AckerleyGiovanni PietrograndeRodolfo GattoGirish C. MelkaniRodrigo RameleGisella VetereRodrigues CileneGiulia CartocciRoger E. StevensonGiulia CuriaRoger KobakGiulia PurpuraRoger M. KrzyżewskiGiulio BernardiRoger RamsbottomGiuseppe FenuRohini ThimmaiahGiuseppe ForteRohit JainGiuseppe LucenteRoland PataiGiuseppe MainaRoland PochetGiuseppe MaulucciRoman NowickiGiuseppe PelliccioniRomina RomanielloGiuseppe SignorielloRonald GillamGlenn BakerRonan MurphyGniewko WięckiewiczRondy YuGonzalo MarquezRoopma WadhwaGonzalo Varas-DíazRory John BufacchiGopinath MuruganandamRosa Angela FabioGoro MaeharaRosa DireitoGraciela Catalina Alatorre-CruzRosa IodiceGrazia SpitoniRosa María Cabanas ValdésGrazyna LietzauRosa VaccaGreg KroliczakRosalia BertorelliGregor VolbergRosario J. MarreroGregory AaenRosario MaugeriGreta MainieriRosario SarabiaGretchen E. TietjenRosemary S. C. HorneGrigorios NasiosRosi AlessiaGrzegorz WojcikRossana RautiGrzegorz WysiadeckiRoy McConkeyGuangli LiRubén Carlos AcevedoGuangting MaiRuben Lopez-BuenoGuglielmo PuglisiRüdiger HeimgärtnerGuido WassinkRüdiger RuppGuifen ChenRudolf KorinthenbergGuillaume HerbetRui LiGuillermo BorragánRuman BanerjeeGunha KimRutvik H. DesaiGunilla BohlinRwei-Ling YuGünther PalmRwik SenGuofang ShenRyan P. M. HackländerGustavo Eduardo TafetRyoki SasakiGustavo Rodríguez FuentesRyouta MatsuuraGuy BosmansRyuji OchiaiGuy TraininS. Iglesias-ParroGvozden RosicSabata PiernoHadas ShintelSabina Barrios-FernándezHaewon ByeonSabina KrupaHahn Young KimSabina VatterHaichao WeiSabine PlancoulaineHamdi ChtourouSabrina M. Di Lonardo BurrHamid CharkhkarSadegh MoradiHamid Reza MaratebSakthivel SrinivasanHan ReichgeltSalman FaruqiHankyu LeeSalman HussainHanna SchleihaufSalvador Castaneda VegaHans H. StassenSalvador Romero-ArenasHans RichterSalvatore MazzeoHanspeter A. MallotSalwa El TawilHany HassaninSam David ParsonsHao LiSamad EsmaeilzadehHaosu ZhangSamantha AbramHarald KindermannSamantha AudrainHaris MemisevicSamantha BartonHarishchandra DubeySamar S. AyacheHarold L. RekateSambuddha BasuHarold MourasSamer K. ElbabaaHarriet BallSami L. KhellaHartmut VatterSami ObaidHaruki KoikeSamuel FrankHarvey SarnatSamuel GreiffHatice KumruSamuel X. ShiHeather F. SmithSanaz PournajafHeather M. HowerSandeep DaveHeather N. RichardsonSandeep Kumar BarodiaHéctor García- LópezSandra MartinsHeechul YoonSandra McFaddenHeidi HaavikSandra PellizzoniHeidi MeyerSandro OrrùHeike ArgstatterSang Ouk WeeHeiko BrennenstuhlSang-Mok LeeHelané WahbehSantiago CepedaHelen Anne LeaverSantiago HaaseHelen FillmoreSara Anna BoniniHelene A. FachimSara Bandres-CigaHelene FachimSara BertoniHelga MiguelSara BorgomaneriHelmar Bornemann-CimentiSara GuedicheHelmut FrohnhofenSara InvittoHelmut Schrom-FeiertagSara ParmigianiHemendra VekariaSara PerettiHenk J. EilanderSara RinaldiHenrietta NittbySara Scharoun BensonHenrik DruidSara SommarivaHenry SutantoSarah HellewellHermann HinrichsSarah JessenHernan Gonzalo ReySarah Kittel-SchneiderHerve PlatelSarah L. KaralunasHideaki InagakiSarah M. Dos AnjosHidenori OgataSarah MeyerHideto NakajimaSarath VijayakumarHidetoshi KomatsuSatja Mulej BratecHila Zahava GvirtsSaúl Gómez-ManzoHilmar SigurdssonSaulius LukoseviciusHimashu Narayan SinghSaurabh SonkusareHipólito MarreroSaurabh SrivastavaHipolito NzwaloScavuzzo ClaireHiroharu KataokaSchintu SeleneHiroyuki SawatariScott SpreatHisham BahmadSD RabkinHohyun ChoSe Jin ParkHojong ChoiSean MeehanHon Fai ChanSebastian FrankeHongfang WangSebastian SchadeHongwu WangSebastian SchindlerHouman SotoudehSebastiano TorrisiHsin-chi KoSébastien CarnicellaHubertus HimmerichSeikwan OhHui-Rong JiangSelene SchintuHung-Pin TuSelina Christin WriessneggerHunter GroningerSelma StaegeHye Mi KwonSelma SupekHye-geum KimSeokhoon YoonHye‐Kyoung KimSeoung Hoon ParkHyemin HanSepehr SaniHyeon Kyu LeeSeppo AhlforsHyungon LeeSerena DattolaIbai DiezSerge-Daniel Le BonIdan TalSergei G. GaidinIgnacio ArreseSergei L. ShishkinIgnatius NipSergey KolomeǐchukIgnazio Gaspare VetranoSergey KornilovIgor JakovcevskiSergio BagnatoIldiko RaczSergio Lerma LaraIlona KopytaSergio Muniz-CastrilloIlona PapousekSerra MarcelloIlona Rubi-FessenSeverin SchrammIman BeheshtiSeyma KatrinliImme LammertinkSezgi GoksanIndrani PoddarShahab HaghayeghInga Griskova-BulanovaShaina PonceIngrid H. C. H. M. PhilippensShalaka MulherkarIoannis VamvakarisShalini NarayanaIole PintoShane FresnozaI-Ping HsuehShanmugarajan KrishnanIppei NojimaShaoqiu HeIrena Baranowska-BociaskaSharon AshIrena RektorovaSharon Morein-ZamirIrene De BoerShawn FlanaganIrene Santos-BarriopedroSheila BlackIrene Torres-SánchezSheina EmraniIrina NovikovaShenghong HeIris Garcia-MartínezShengwen Calvin LiIris HaimovSherain HarricharanIryna BabikShigeo OhbaIryna RusanovaShigeru YokoyamaIsa EbtehajShikha PrashadIsabel C.H. ClareShin-Ichi IzumiIsabel Esteban-RodríguezShinji KakeiIsabel Garcia-GarciaShiri Lev-AriIsabel María Martín MonzónShirley WyverIsabel Varela-NietoShivam Om MittalIsabell CordtsShovan NaskarIsabella ZanellaShunta MaedaIsao NambuShweta GuptaIsmael Martínez-GuardadoSian Andrea WilliamsIvan BrakSiddhita MhatreIvan Miguel PiresSigan L. HartleyIvan N. PigarevSilke GöbelIvan Sanchez-FernandezSílvia LopesIvan Y. IourovSilvia Martinez FerreiroIwona Piatkowska-ChmielSilvia MiddeiJ. Gavin BremnerSilvia PixnerJ. Scott YarussSilvia PrimativoJ. Simon WiegertSilvia SondereggerJaanus HarroSilvio IontaJacek BucznySimon Morand-BeaulieuJacek RogalaSimon PodnarJack RogersSimona CabibJaclyn DyniaSimona LucibelloJacobo Fernandez-VargasSimona MitevaJacopo Junio Valerio BrancaSimona NicoaraJacopo LamannaSimona SacchiniJacqueline CummineSimona VecchiJacqueline LeventonSimone BattagliaJacques LautreySimone GastaldonJacques-Olivier CoqSimone GillJade CartwrightSimone RossiJaesok YuSina ShadfarJae-Sung YooSireesha ManneJaeyong ParkSiri-Maria KampJakob Åsberg JohnelsSirwan DarweeshJames A. HendrixSivaraj Mohana SundaramJames BrasicSiwei ZhangJames E. Jr. HassellSiyuan GaoJames F. PagelSlaven PikijaJames HendrixSlawomir TobisJames KirbySobh ChahbounJames NegenSofia CuocoJames P. TrujilloSofia HadjileontiadouJames StaffordSoledad BallesterosJames T. MantellSolveig Lægreid HaugerJames W. SonneSomayeh AghanavesiJamie M. BogleSonia DarbraJamir Pitton RissardoSonja KoskiJan BroggerSophie DuportJan FayeSotirios ApostolakisJan J. ZebrowskiSouad Khellat KihelJan Manuel Rodriguez ParkitnaSoumi KunduJan P. RöerSpyridon SiafisJan PurruckerSreenath NairJan StoopStacey L. RobinsonJana Ruda-KucerovaStave KohtzJana TegelbeckersStefan A. CarpJanani IyerStefan DumaJanani ParameswaranStefan KammermeierJane JöhrStefan PuschJanek LobmaierStefan RamppJanet M. Menzie-SuderamStefan WederJanet S. DufekStefan ZirnJanice ForsterStefania ToselliJanna DickensonStefanie FlunkertJannick NiortStefanie WillekensJannik PrasuhnStefano LasaponaraJanusz BłaszczykStefano Maria PriolaJanusz SobeckiStefano TozzaJared BengeStella María Fuensalida NovoJarkko JohanssonStepan CapekJaromír MyslivečekStephan PaulJasneek K. ChawlaStephanie G. B. SullivanJason C. ParkStéphanie LefebvreJason S. RadowskyStephanie N.L. SchmidtJasper O. NuningaStephen KanneJavier Pérez FernándezStephen MullinJavier Quillo-OlveraStephen SchultzJavier RaseroSteven D. ShirkJavier SantabarbaraStormi Pulver WhiteJavier VilladiegoStyliani VlachouJay S. MishraSue BuckleyJean A. RondalSuelen BoschenJean-François PicimbonSujoy Ghosh HajraJean-Michel EscoffreSumitha Rajendra RaoJeanne GalléeSummer SheremataJean-Paul NoelSun ImJeff GibbonsSunan LiJeffery HainesSungmin ChoJeffrey HubbardSunil SirohiJeffrey M. EngelmannSuresh NeethirajanJeffrey W. BellSusan FranksJelena HyppönenSusan LoveallJeng-Ren DuannSusan SaraJenni RediferSusana PintoJennifer FanningSusanna SummaJennifer LaCosseSusanne OhmannJennifer R. Stapleton-KotloskiSuzanne HanserJennifer RyanSuzanne Perea BurnsJennifer T. LambertsSy Duong-QuyJennifer VonkTadashi ItoJenny C. A. ReadTakayasu MishimaJenny Van Der SteenTakayuki KatayamaJens GaabTeikichi IkuraJens-Ulrich RahfeldTeo KejiaJeppe LangeTetsufumi ItoJérémie SellamThammanard CharernboonJeremy HarperTommaso ErcoliJeroen A. Van WaardeTomoyo MoritaJeroen Van SchependomTomoyuki KuwakiJérôme CarriotTony J. CunninghamJérôme CosteToyoaki OhbuchiJeronimo AuzmendiTrent R. AndersonJerónimo GonzálezTurcu-Stiolica AdinaJerry HoepnerUday PratapJesper Just FabriciusUrsula SandauJesse L. KowalskiUrvish PatelJesse R. SchankVaitsa GiannouliJesse SargentValeriia DemarevaJessica A. SternVasileios ChristouJessica BurketVera GramignaJessica PeterVigouroux NadineJessica W. YoungerVittorio Emanuele BianchiJesus Cruz-GarzaVivek PecheJesus PastorVladimir KenisJesús PlanagumàVojtěch JuříkJia GuoVyacheslav KarolisJia LuoWakiro SatoJiachen ZhuoWalter P. PflieglerJianxiong JiangWeihua YueJiawei WangWeili LuJill E. LarsonWilliam BergJill LobbestaelWilliam Clay JacksonJill R. CrittendenWilliam GrishamJillian Vinall MillerWinko W. AnJing FanWolfgang BergerJingjing ZhaoWoon-Man KungJiri PopelarXiphias ZhuJiro ShimayaXiuyun LiuJitendra SinghYasunari KandaJitka AnnenYoav KesslerJitka Třebická FialováYonata LevyJo SourbronYoshiaki AdachiJoan DuprezYosky KataokaJoana CamposYoung Chul YounJoana DuarteYuan ZhangJoanna ListosYun-bae KimJoanna PawlakYutaka OwariJoanna SchugZafer KeserJoanna SolichZargari Marandi RamtinJoão CastelhanoZeeshan Ahmad KhanJoao PeçaZsuzsanna MihályJochen Kaiser



